# Effects of Flavonoids from Food and Dietary Supplements on Glial and Glioblastoma Multiforme Cells

**DOI:** 10.3390/molecules201019406

**Published:** 2015-10-23

**Authors:** Marko Vidak, Damjana Rozman, Radovan Komel

**Affiliations:** Institute of Biochemistry, Faculty of Medicine, University of Ljubljana, Vrazov Trg 2, SI-1000 Ljubljana, Slovenia; E-Mails: damjana.rozman@mf.uni-lj.si (D.R.); radovan.komel@mf.uni-lj.si (R.K.)

**Keywords:** food, dietary supplements, flavonoids, quercetin, catechins, proanthocyanidins, bioavailability, blood-brain barrier, glial cells, glioblastoma multiforme

## Abstract

Quercetin, catechins and proanthocyanidins are flavonoids that are prominently featured in foodstuffs and dietary supplements, and may possess anti-carcinogenic activity. Glioblastoma multiforme is the most dangerous form of glioma, a malignancy of the brain connective tissue. This review assesses molecular structures of these flavonoids, their importance as components of diet and dietary supplements, their bioavailability and ability to cross the blood-brain barrier, their reported beneficial health effects, and their effects on non-malignant glial as well as glioblastoma tumor cells. The reviewed flavonoids appear to protect glial cells via reduction of oxidative stress, while some also attenuate glutamate-induced excitotoxicity and reduce neuroinflammation. Most of the reviewed flavonoids inhibit proliferation of glioblastoma cells and induce their death. Moreover, some of them inhibit pro-oncogene signaling pathways and intensify the effect of conventional anti-cancer therapies. However, most of these anti-glioblastoma effects have only been observed *in vitro* or in animal models. Due to limited ability of the reviewed flavonoids to access the brain, their normal dietary intake is likely insufficient to produce significant anti-cancer effects in this organ, and supplementation is needed.

## 1. Introduction

Gliomas are malignant tumors of glia, the brain connective tissue. They are ranged from the least severe grade, I, to the most severe grade, IV. Low-grade gliomas (I and II) consist of well-differentiated, slowly proliferating cells while high-grade tumor cells (III and IV) are characterized by their invasiveness and lack of differentiation [1]. Glioblastoma multiforme (GBM) is a type of grade IV glioma notorious for its aggressive clinical manifestation and fatal outcome. Median survival time of GBM patients after diagnosis is only 15 months despite surgical treatment combined with aggressive chemotherapy [2]. In almost all cases, resections fail to remove tumor cells in total, and the cells located in perivascular niches on tumor edges are most likely to be left behind [3]. Many of these cells possess stem-cell like properties such as partial pluripotency, non-differentiated state and self-renewal ability, thus they represent a focal point of new tumor growth [3,4]. Compared with a standard therapy (resection and adjuvant radiotherapy), adjuvant chemotherapy with an alkylating agent temozolomide is unable to extend life for more than two and a half months on average because it is not specifically directed at these stem-like tumor cells [5,6]. GBM tumors readily invade adjacent healthy tissue and metastasize to distant brain regions, but their dissemination outside the brain is usually prevented by the blood-brain barrier (BBB) [7]. Nevertheless, their dissemination within the brain leads to a fatal outcome since it induces brain herniation that disables vital brain centers [8].

Exposure of the brain to oxidative stress is one of the most important risk factors for GBM [9]. It can be mitigated by intake of antioxidants such as flavonoids, which are a class of polyphenolic compounds synthesized naturally by plants [10]. In developed countries, flavonoids can be found both in normal diet and dietary supplements [11]. The aim of this paper is to review the effects of flavonoids on normal glial and malignant GBM cells, with a focus on flavonoids that frequently appear in food and dietary supplements: quercetin, proanthocyanidins, and catechin derivatives (*i.e.*, catechin, epicatechin, epigallocatechin, epicatechin gallate and epigallocatechin gallate). These compounds have been chosen to be reviewed in detail because they are present in common foodstuffs and readily available as supplements.

In the first part of the review, a general molecular structure of flavonoids will be described, followed by their classification into types and assessment of their bioavailability and ability to cross the BBB. In the second part, quercetin, catechins and proanthocyanidins will be reviewed by discussing their prevalence in diet and dietary supplements, reported beneficial effects on health, and effects on glial and GBM cells.

## 2. Flavonoids

Flavonoids are colored polyphenolic compounds that appear naturally in plants as secondary metabolites. Their main roles in plant physiology are protection against ultraviolet radiation and attraction of pollinating insects [12]. Their molecular structure is characterized by a chromane heterocyclic ring with a phenyl substituent at C-2. Flavonoids are divided into several classes and groups based on substituents at C-3 and C-4, and presence of a double bond between C-2 and C-3 (Table 1). Naturally occurring plant flavonoids often form dimers and oligomers, as well as esters with gallic acid (gallates) or ethers with carbon hydrates (glycosides) [13].

**Table 1:** Classification and structural formulae of flavonoids.

Chromane Ring					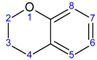	
General Structural Formula of Flavonoids (2-Phenylchromane)					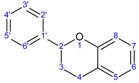	
Class	Group	C2-C3 Double Bond	3-OH	4-keto	Structural Formula	Example
**Anthoxanthins**	Flavones	Yes	No	Yes	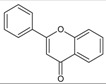	Apigenin, luteolin.
	Flavonols	Yes	Yes	Yes	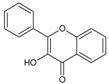	Quercetin, kaempferol
**Flavanones**	/	No	No	Yes	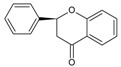	Naringenin
**Flavanonols**	/	No	Yes	Yes	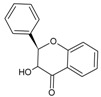	Taxifolin
**Flavans**	Flavan-3-ols	No	Yes	No	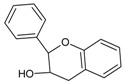	Catechin and its derivatives.
	Flavan-4-ols	No	No (4-OH instead)	No	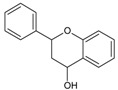	Apiforol
	Flavan-3,4-diols	No	Yes (also 4-OH)	No	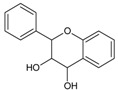	Leucocyanidin
	Proanthocyanidins	No	Depending on monomers.	No	Figure 3	Dimers and oligomers of flavanols
**Anthocyanidins**	/	No (aromatic ring)	Yes	No	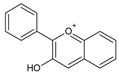	Cyanidin

The main absorption site of dietary flavonoids is the small intestine where they mainly appear as glycosides, which are bulky and polar molecules with low permeability coefficients [14]. However, their glycoside moieties may bind to the sodium-glucose transport protein 1 (SGLT 1), which is a type of glucose transporter [15]. Flavonoid glycosides are thus transported in enterocytes where they undergo cleavage of glycoside bond and conjugation with glucoronate, methyl or sulfate groups. Conjugated and unconjugated flavonoids are absorbed from enterocytes into portal circulation. Unconjugated molecules are extensively metabolized during their first pass through the liver, so that the bulk of plasma flavonoids are conjugates [16]. In enterocytes, flavonoids are also substrates for efflux transporters that transport them back to intestinal lumen [17], further limiting their bioavailability.

Flavonoids can cross the BBB with transcellular diffusion, carrier-mediated transcellular transport or paracellular diffusion through tight junctions between the endothelial cells of the BBB [18]. For both types of diffusion, molecular size is a limiting factor favoring smaller flavonoid molecules such as unconjugated monomers. Passive transcellular diffusion is additionally limited to small molecules with sufficient lipophilicity [19]. Conjugates and oligomers can enter the brain mainly with the help of transporters such as organic anion-transporting polypeptides (OATPs) [20]. However, carrier-mediated transport across the BBB is bidirectional because flavonoids are substrates for P-glycoprotein (P-gp) [21], an efflux transporter that carries substrate molecules from the brain interstitial fluid (ISF) to endothelium [22]. Since all brain regions are not equally perfused with ISF, flavonoids tend to concentrate in more perfused regions [21]. To exert their effects, ISF flavonoids have to enter neurons or glial cells, thus their presence in ISF is not necessarily in correlation with their effects [18]. ISF has a high turnover rate and is continuously secreted across the BBB [20], carrying contained flavonoids with it.

The main systemic action of flavonoids is reduction of oxidative stress. It is mediated by five mechanisms—direct scavenging of reactive oxygen species (ROS) and reactive nitrogen species (RNS), prevention of Ca^2+^ influx despite increased ROS/RNS levels, increase in the levels of endogenous ROS/RNS scavengers such as glutathione (GSH) [23], alteration of mitochondrial function [24], and decrease in the expression of enzymes involved in ROS/RNS generation such as nitric oxide synthase (NOS) [25]. Reduction of oxidative stress results in neuroprotective and anticarcinogenic activity [24,26]. Antioxidative action of flavonoids supplements that of endogenous ROS scavengers, e.g., ascorbic acid, tocopherols, GSH, thioredoxin and glutaredoxin. ROS are also neutralized by enzymes—catalase, peroxidases and superoxide dismutases (SOD)—which convert them into less reactive compounds. In addition to flavonoids, there are other plant-derived ROS scavengers such as lignin precursors and tannins [27,28].

Other reported beneficial effects of flavonoids include reduction of inflammation due to suppressed expression of cyclooxygenase 2 (COX-2) [25], modulation of efflux proteins such as multidrug resistance-associated proteins (MRPs) [29], inhibition of phase I metabolic enzymes and induction of phase II enzymes [26], inhibition of DNA topoisomerases [30], induction of apoptosis [31], and inhibition of protein kinases involved in proliferative signal transduction [26,32]. Effects of flavonoids on signaling pathways are summarized in Table 2.

Studies in rodents have reported lack of toxicity of flavonoids even in cases of overdose [33,34]. However, flavonoids have a few adverse effects. In high concentrations, flavonoids may promote oxidation even though they otherwise act as antioxidants [35]. If consumed excessively during pregnancy, they may disrupt fetal development, mainly due to their inhibition of DNA topoisomerases, which are highly expressed in fetus [36]. A flavonol myricetin and its 3-galactoside have pronounced cytotoxic effects caused by their inhibition of topoisomerase I [30], while quercetin-mediated inhibition of fetal topoisomerase II results in increased risk of infant leukemia [36]. A flavonon naringenin is highly teratogenic and often lethal for fetus in animal models. Surviving fetuses suffer from developmental retardation and defects of neural tube closure [37]. Other adverse effects of flavonoids include liver damage and contact dermatitis [26]. Flavonoids may also interact with other herbal compounds, as well as with prescribed drugs, altering their bioavailability and therapeutic effectiveness [38].

**Table 2:** Signaling effects of flavonoids.

		Signaling Pathways (Red = Pathway Inhibition, Green = Pathway Activation)											
Group	Flavonoid	Ras/MAPK	EGF/PI3K/Akt	NF-κB	Wnt/β-catenin	TNFα/NADPH-oxidase	JAK/STAT	Notch	ER	AHR	Nrf2	Ig-E	IRF-1
Flavonols	Quercetin												
	Kaempferol												
	3′-HF												
Flavan-3-ols	Catechin/CG												
	Epicatechin												
	Epicatechin metabolites												
	ECG												
	EGCG												
PACs	Dimeric procyanidins												
	Hexameric procyanidins												
Flavones	Apigenin												
	Luteolin												
	Tangeretin												

References: [39,40,41,42,43,44,45,46,47]; Abbreviations—Signaling pathways: MAPK = mitogen-activated protein kinase, EGF = epidermal growth factor, PI3K = phosphatidylinositide-3-kinase, Wnt = wingless-related integration site, NF-κB = nuclear factor kappa B, TNFα = tumor necrosis factor alpha, NADPH = nicotinamide adenine dinucleotide phosphate, JAK = Janus kinase, STAT = signal transducer and activator of transcription, ER = estrogen receptor, AHR = aryl hydrocarbon receptor, Nrf2 = nuclear factor 2, Ig-E = immunoglobulin E, IRF-1 = interferon regulatory factor 1; Flavonoids/groups: 3′-HF = 3′-hydroxyflavone, CG = catechin gallate, ECG = epicatechin gallate, EGCG = epigallocatechin gallate, PACs = proanthocyanidins.

## 3. Quercetin

**Figure 1:**
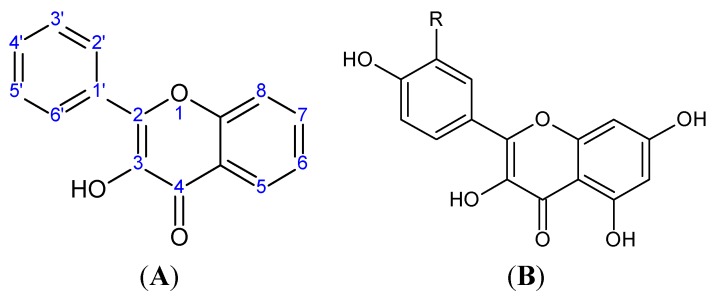
(**A**) General structural formula of flavonols; (**B**) structural formula of quercetin (R = OH) and kaempferol (R = H).

Quercetin is a member of the class of flavonols (Figure 1), which is characterized by a phenyl substituent at C-2, hydroxyl group at C-3, keto group at C-4 and double bond between C-2 and C-3 [48].

Quercetin derivatives are the most abundant flavonoids in Western diet [49] and pure quercetin is also a common ingredient of dietary supplements [50]. Quercetin appears in food mostly in the form of glycoside derivatives such as rutin and quercitrin. Quercetin-containing fruits and vegetables commonly appearing in Western diet are apples, lemons, lettuce, red grapes, cabbage, tomatoes, onions, parsley, pears, plums, cherries, strawberries, blueberries and cranberries, while the richest sources of quercetin are capers, elderberries and lovage leaves [51,52]. Quercetin is also present in black and green tea, red wine, cocoa and olive oil [51]. To achieve quercetin plasma concentration necessary for beneficiary health effects, regular intake of quercetin-rich food is not sufficient [53], thus dietary supplements are needed [54]. Bioavailability of pure quercetin from these supplements is limited by its low solubility in intestinal juice [55], which inhibits its release from a dosage form [56]. On the other hand, larger and more hydrophilic quercetin glycosides are more soluble in intestinal juice but their absorption into enterocytes depends on their carbohydrate moiety. According to Hollman *et al.* [57], after oral administration, the shares of absorbed rutin (quercetin conjugated with the disaccharide rutinose), quercetin glucosides from onions, and quercetin aglycone equal 17%, 52% and 24%, respectively. The difference in absorption of glycosides likely occurs due to SGLT transporters, which facilitate the absorption of glucosides. While its lipophilicity facilitates its diffusion across the BBB, quercetin is also a substrate for P-gp efflux carriers in the BBB [21], which remove it from the brain and lower its net permeability.

Among flavonoids, quercetin is the greatest scavenger of ROS and RNS [58]. Its antioxidant capacity is approximately six times higher than that of ascorbic acid [59]. Its protective effect against oxidative stress results in beneficial effects on the heart and lungs, which help reduce risks of lung cancer [60], asthma [61] and coronary artery diseases [62]. ROS are also involved in promotion of inflammatory processes since they induce the production of cytokines such as tumor necrosis factor alpha (TNF-α) via activation of transcription factors nuclear factor kappa-B (NF-κB) and activator protein 1 (AP-1) [63,64]. By scavenging ROS, quercetin inhibits cytokine production in the lungs [65], brain [66] and macrophages [67], and therefore reduces inflammation. Studies performed with cell lines have shown that quercetin possesses anti-fibrotic [68], anti-bacterial [69], anti-coagulative [70], anti-atherogenic [71], anti-hypertensive [72] and anti-proliferative effects [73,74]. However, most of those effects have not yet been confirmed by epidemiological studies [58].

Protective effects of quercetin on glial cells have been extensively studied, mostly by using C6 cells and astrocyte cultures as models for *in vitro* experiments. Exposure of C6 cells to oxidative stress results in lipid peroxidation, glutathione (GSH) depletion, increased breaking of DNA strains, and increased influx of calcium ions. However, these adverse effects are mitigated if cells are treated with quercetin [75]. Anti-inflammatory effects of quercetin on C6 cells have also been reported. In this cell line, quercetin decreases the expression of cytokines TNFα and interleukin (IL) 1α. Consequent inhibition of inflammatory processes leads to diminished apoptosis of neurons [66]. In addition, quercetin inhibits apoptosis of C6 cells via induction of heme oxygenase 1 (HO-1), a protein belonging to the family of heat shock proteins (HSPs) [76]. Effects of quercetin on HSPs are not uniform and there are cases in which this flavonoid acts as an inhibitor. For instance, in primary culture of astrocytes, quercetin inhibits expression of three HSPs—c-fos protein, HSP70 and glial fibrillary acidic protein (GFAP)—which are involved in injury response. Their expression leads to the formation of an astrocytic scar that replaces injured neurons and represents a hallmark of brain damage [77]. Quercetin may therefore reduce the extent of brain damage caused by trauma. According to Panickar *et al.* [78], in C6 cells quercetin reduces ischemia-induced cell swelling following brain trauma. It also attenuates two other processes that accompany ischemic injury and are implicated in brain edema, namely the increase in intracellular calcium ions and production of free radicals. However, Volk *et al.* [79] reported that quercetin inhibited lactate transport in C6 cells and rat astrocytes, causing a significant accumulation of intracellular lactate, decrease of intracellular pH, and cell swelling due to increased osmotic pressure caused by lactate accumulation.

Despite inhibiting apoptosis of neurons and C6 glial cells, quercetin induces apoptosis in human glioblastoma multiforme T98G cells by activating the mitochondrial death pathway. Exposure of T98G cells to quercetin leads to activation of caspases 3 and 9, release of cytochrome c from the mitochondrium and a decrease in the mitochondrial membrane potential. Quercetin also inhibits heat shock proteins HSP27 and HSP72, which are involved in the mitochondrial apoptotic pathway [80]. Braganhol *et al.* [81] compared quercetin effects in the U138MG glioma cell line and hippocampal organotypic cultures. In U138MG cells, quercetin decreased cell proliferation and viability, induced necrotic and apoptotic cell death, arrested the cell cycle and decreased the mitotic index, while it prolonged the survival of hippocampal cells in the face of ischemic damage. Kim *et al.* [82] reported that treatment of human A172 glioma cells with quercetin caused rapid reduction in phosphorylation of extracellular signal-regulated kinase (ERK) and Akt, resulting in decreased cell viability. Moreover, quercetin increased glioma cell apoptosis by stimulating caspase activity and decreasing expression of survivin, an antiapoptotic protein. In a study by Siegelin *et al.* [83], quercetin exposure resulted in proteasomal degradation of survivin in the A172, U87-MG and U251 cell lines, but not in U373 glioma cells. Quercetin-induced degradation of survivin increased apoptosis mediated by TNF-related apoptosis-inducing ligand (TRAIL). However, Kim *et al.* [84] reported that quercetin-induced apoptosis in U373 cells by increasing the expression of tumor suppressor protein p53, which in turn released cytochrome c from mitochondria to the cytosol, resulting in the activation of the mitochondrial pathway. According to this study, quercetin also induces protective autophagy. This finding has been challenged by Jakubowicz-Gil *et al.* [80], who found no connection between quercetin and autophagy in T98G cells, while in the grade III astrocytoma cell line MOGGCCM quercetin stimulated autophagy only when combined with a low dose of temozolomide [85]. In T98G and U87 glioblastoma cells, quercetin inhibits the release of IL-6, an important cancer-related cytokine, which in turn activates the pro-oncogene STAT3 signaling pathway. In addition, the quercetin-mediated inhibition of IL-6 decreases proliferative and migratory properties of glioblastoma cells. Quercetin also modulates the expression of two target genes regulated by STAT3, *i.e.*, cyclin D1 and matrix metalloproteinase 2 (MMP-2) [86]. Isoquercitrin, a glycosylated derivative of quercetin, inhibits proliferation of glioblastoma cells via reduction in Wnt/β-catenin signaling activity [87].

Kaempferol is a flavonol closely structurally related to quercetin, from which it is distinguished only by its lack of a hydroxyl group at C-3′ (Figure 1). Several mechanisms of its anti-glioma activity have been reported. Kaempferol facilitates apoptosis of glioma cells by reducing levels of anti-apoptotic protein survivin and sensitizing the cells to the effects of tumor necrosis factor-related apoptosis-inducing ligand (TRAIL) [88]. It also suppresses migration and invasion of GBM8401 glioblastoma cells by blocking pro-oncogene signaling cascades mediated by mitogen-activated protein kinase (MAPK), NF-κB and Akt [89] (Table 2).

Flavones differ from flavonols by lacking a hydroxyl group at C-3 (Table 1). Luteolin, apigenin and hispidulin are examples of flavones with anti-glioma activity. Luteolin suppresses migration of GBM8401 cells in a manner similar to that of kaempferol, described above [89]. Apigenin triggers apoptosis of human glioblastoma cells T98G and U87MG, without affecting non-malignant human astrocytes [90]. Hispidulin inhibits proliferation of GBM cells by inducing their apoptosis and arresting cell cycles [91].

## 4. Catechins

**Figure 2:**
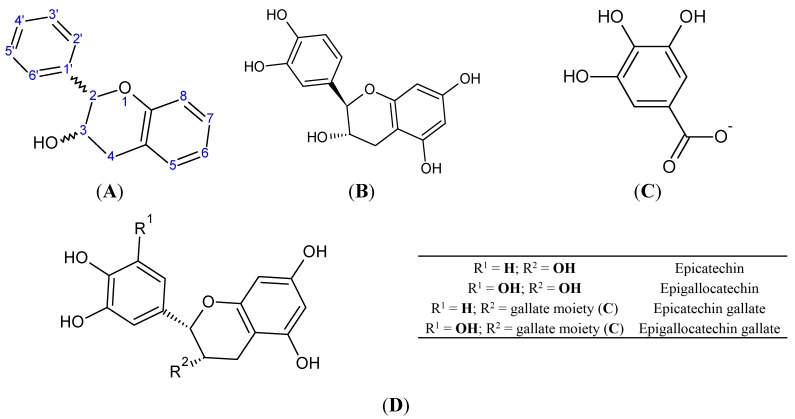
(**A**) General structural formula of flavan-3-ols; (**B**) (+)-catechin (2*R*,3*S*), the more common of the two catechin enantiomers; (**C**) gallate moiety; (**D**) Substituents of catechin derivatives.

Catechins are derivatives of the eponymous flavonoid catechin, which belongs to the class of flavan-3-ols (Figure 2), characterized by a single bond between C-2 and C-3, dihydroxyphenyl substituent at C-2, hydroxyl group at C-3, and lack of a keto group at C-4 [92]. Catechin derivatives commonly found in food and dietary supplements are epicatechin (EC) and epigallocatechin (EGC), as well as their respective esters with gallic acid: epicatechin gallate (ECG) and epigallocatechin gallate (EGCG) (Figure 2). Epicatechin is a common name for the two diastereoisomers of catechin, while the name epigallocatechin is used for the diastereoisomers of gallocatechin, a catechin derivative with a trihydroxyphenyl group at C-2. ECG and EGCG have a gallate moiety attached at the C-3 hydroxyl group [93,94]. All the catechin derivatives may appear in food in the form of glycosides [95].

The arguably most important dietary source of catechins is cocoa and its derivatives such as chocolate [96]. Other fruits, vegetables and beverages with high content of catechins are black and green tea, red wine, broad beans, custard apples, strawberry tree fruits, apples, peaches, cherries and plums [97,98,99]. Isolated green tea catechins are also available as dietary supplements [100]. Pharmacokinetics of catechins is characterized by their low bioavailability, caused by slow absorption, extensive first pass metabolism and wide tissue distribution [101,102]. The most common metabolites of catechins are glucoronate conjugates [103]. However, the both gallates (ECG and EGCG) are less extensively metabolized during their absorption in the systemic circulation, thus they are found in the bloodstream mostly in their original forms [14,104]. In enterocytes, catechins are subjected to transporter-mediated efflux, which additionally lowers their bioavailability [105]. Catechins are able to pass the BBB, but during their transport across endothelial cells composing the BBB, they are subjected to metabolic transformations and efflux [18,106]. Their transport efficiency is therefore low: according to Faria *et al.* [106] who used RBE4 cells as a BBB model, 28% of blood epicatechin and 15% of blood catechin is transported to the brain, while according to research in rats performed by Wu *et al.* [107], these percentages are 11% and 7% for epicatechin and catechin, respectively. Catechin glucuronides and gallates are also able to pass the BBB, but their transport efficiency is even lower [106,108].

Catechins are powerful antioxidants and scavengers of ROS and RNS [109,110] but low bioavailability limits their protective effects on internal organs [111]. Nevertheless, after the ingestion of food or dietary supplements containing catechins, unmetabolized forms of these compounds reach sufficiently high local concentrations in the digestive tract to protect cells from oxidative stress and malignant transformation [112]. Consequently, regular ingestion of catechins reduces the risk of digestive tract cancers [113,114,115,116]. In animal models, catechins also inhibit carcinogenesis of liver [117,118], breast [119,120], prostatic [121] and lung cancers [117,122,123]. In addition, they modulate immune response [124], suppress hyperlipidemia [125], prevent hepatotoxicity [126,127] and nephropathy [128,129], and reduce risks of cardiovascular diseases due to their antithrombotic effects [130].

Catechin derivatives exert anti-inflammatory effects in the brain [131]. In glial cells, catechin and EGCG modulate kinase signaling pathways, e.g., by inhibiting the signaling cascade of mitogen-activated protein kinase (MAPK), which regulates the expression of the pro-inflammatory cytokine TNF-alpha and the enzyme inducible nitric oxide synthase (iNOS). Consequently, the release of TNF-alpha and nitric oxide (NO) is reduced, leading to attenuated neuroinflammation [132,133,134]. Catechin and EGCG also reduce prostaglandin synthesis by inhibiting COX-2 expression [135,136], and suppress the generation of ROS and RNS [137,138], thus providing glial cells with additional protection against inflammation and oxidative stress. Panickar *et al.* [139] reported that in C6 cells, a combination of catechin, epicatechin and EGCG attenuated ischemia-induced astrocyte swelling, which is a major component of cytotoxic brain edema. However, each of these catechins was unable to reduce the swelling by itself. Beneficial effects of ECG have also been reported. According to Abib *et al.* [140], ECG increased glutamate uptake into C6 cells and thus attenuated the effects of glutamate on neurons. Referred to as excitotoxicity, these effects are implicated in the pathogenesis of ischemia and neurodegenerative diseases. ECG also increased secretion of S100A9, a calcium-binding protein that exerts neurotrophic effects on neighboring neurons, astrocytes and microglia.

EGCG appears to be the only catechin derivative with significant anti-GBM activity. It inhibits telomerase in the cell lines 1321N1 and U87-MG, and therefore increases sensitivity of tumor cells to anti-cancer therapy with cisplatin and tamoxifen [141]. Chen *et al.* [142] reported that EGCG increased sensitivity of the cell lines U87 and U251 to temozolomide, the drug of choice for GBM chemotherapy. In mice with implanted tumors consisting of these cell lines, EGCG decreases the expression of glucose-regulated protein (GRP) 78, an endoplasmatic reticulum chaperone that contributes to the cancer cell resistance to temozolomide [142]. Moreover, EGCG induces apoptosis of U251 cells via the laminin receptor, decreases their invasiveness by reducing expression of the matrix metalloproteinases MMP-2 and MMP-9, and inhibits their proliferation by modulating the MAPK pathway [143]. EGCG also decreases the proliferation rate of U87 cells and counters the effects of overexpression of the anti-apoptotic protein survivin, which induces resistance against ionizing radiation [144]. According to Agarwal *et al.* [145], EGCG decreased invasiveness of U87-MG cells, induced their apoptosis and downregulated the levels of pro-inflammatory cytokines. The authors suggested that EGCG induced apoptosis by elevating oxidative stress via ROS generation, but this conclusion contradicts an established view of catechins as ROS scavengers [137]. In human brain microvascular endothelial cells, EGCG intensifies the effects of ionizing radiation. These effects comprise stimulation of cell necrosis and expression of CDK inhibitors p21 and p27. On the other hand, EGCG does not induce the pro-apoptotic proteins caspase-3, caspase-9 and cytochrome C in microvascular endothelial cells [146]. In GBM, these cells are adjacent to perivascular niches, which contain therapy-resistant glioblastoma stem cells [3]. EGCG therefore represents a potential agent for necrosis induction in perivascular niches.

## 5. Proanthocyanidins

**Figure 3:**
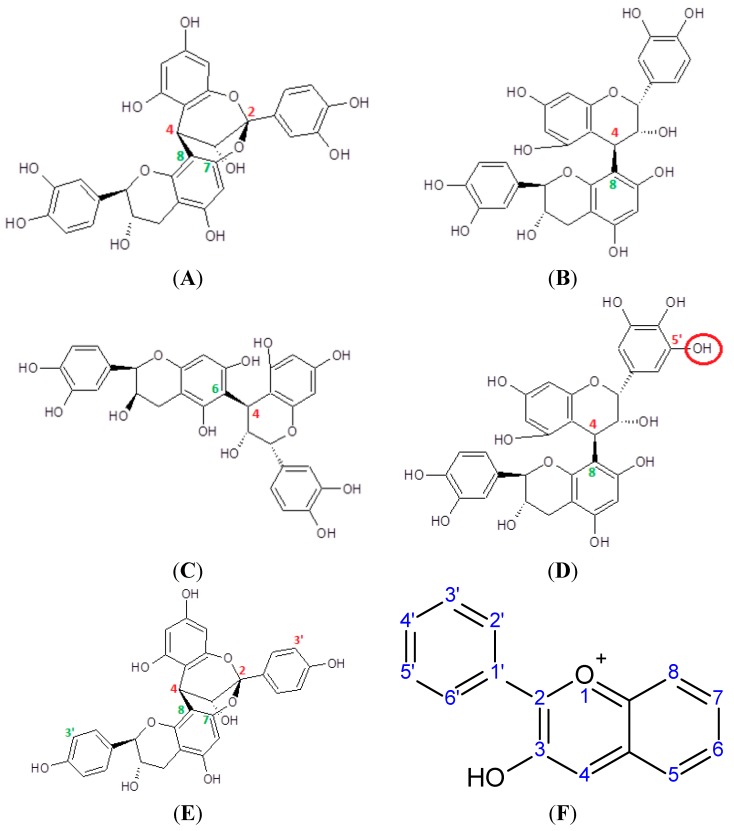
(**A**) Dimeric A type procyanidin (epicatechin-catechin dimer) with C-4→C-8 linkage and C-2→O→C-7 ether bond. Red numbering: epicatechin moiety; green: catechin moiety; (**B**) dimeric B type procyanidin (epicatechin-catechin) with C-4→C-8 linkage. Red: epicatechin moiety; green: catechin moiety; (**C**) dimeric B type procyanidin (epicatechin-epicatechin) with C-4→C-6 linkage; (**D**) dimeric B type prodelphinidin (EGC-catechin) with C-4→C-8 linkage. Red: EGC; green: catechin. Additional OH group at 5′ is marked; (**E**) dimeric A type propelargonidin (epiafzelechin-afzelechin) with C-4→C-8 and C-2→O→C-7 bonds. Red: epiafzelechin; green: afzelechin. Note the lack of OH groups at the both 3′ positions; (**F**) general structural formula of anthocyanidins.

Molecules of flavan-3-ols can form C-C and ether bonds with each other, resulting in the formation of polymers and oligomers collectively referred to as proanthocyanidins [147]. Number of monomeric units represents the degree of polymerization (DP) [148]. Oligomers are characterized by DP ≤ 10, while polymers have DP > 10 [149]. Monomeric units are mainly linked with C-4→C-8 bonds (Figure 3), but the C-4→C-6 linkage also exists. An additional ether bond can be formed between C-2 and the hydroxyl group at C-7, complementing the C-C bond (Figure 3). Flavan-3-ols that figure as monomers in proanthocyanidins are the catechin derivatives discussed in the previous heading, as well as afzelechin and epiafzelechin. The latter two are characterized by a p-hydroxyphenyl substituent at C-2. Polymers and oligomers consisting of exclusively catechin, epicatechin and ECG belong to the subclass of procyanidins, while the proanthocyanidins that also contain gallocatechin/EGC/EGCG or afzelechin/epiafzelechin as monomer units are respectively designated as prodelphinidins and propelargonidins. Procyanidins are frequently found in plants while the other two subclasses are less common in nature [148,150]. All proanthocyanidins can undergo oxidative cleavage, which produces cationic monomers known as anthocyanidins [151].

Many widely consumed fruits, vegetables and beverages contain both proanthocyanidin oligomers and polymers, for example, apples, grapes, wine, plums, apricots, green pears, peaches, blueberries, strawberries, black currant, cocoa, several sorts of beans, almonds, hazelnuts and pistachios. In some cases, only oligomers are present, such as in cherries, bananas, mangos, avocados, kiwi fruits, peanuts and beer [149,152]. An extract produced from the bark of maritime pine contains 65%–75% proanthocyanidins, which have their DPs between 2 and 12. This extract is commercially available as a dietary supplement [153,154]. The grape seed extract (GSE) is another example of a supplement rich in proanthocyanidins [155].

Human gastric acid and enzymes cannot metabolize proanthocyanidins into their monomeric units [156], and the bioavailability of unmetabolized oligomers and polymers is very low [14]. However, intestinal bacteria can metabolize some of these compounds. The products of bacterial metabolism are phenolic acids such as phenylacetic, phenylpropionic, phenylvaleric and benzoic acid [157,158], which are absorbed easier than unmetabolized proanthocyanidins [159]. Lactons of phenolic acids can also be formed [160]. According to Deprez *et al.* [161], proanthocyanidin dimers and trimers can be absorbed from the intestinal lumen in a significant extent, likely via paracellular diffusion, while the absorption of polymers is negligible. Absorbed proanthocyanidins are subjected to *O*-methylation by the enzyme catechol-*O*-methyl transferase (COMT) [162]. Enzymes in enterocytes are also likely responsible for cleaving oligomers into their monomeric units, as well as for the conjugation and methylation of resulting monomers [162,163]. However, in a study by Donovan *et al.* [164], no procyanidins were detected in plasma after oral administration of procyanidin dimers to rats. To our best knowledge, there has been only one report about proanthocyanidins being able to cross the BBB [165]. On the other hand, Robert *et al.* [166] reported that proanthocyanidin oligomers decreased the permeability of BBB. Metabolism of proanthocyanidins produces monomeric catechins that are able to penetrate into the brain. Prasain *et al.* [167] conclusively detected catechin and epicatechin in the brain of rats fed with GSE.

The main beneficial health effect of proanthocyanidins is their antioxidative activity [150,168]. Results of human clinical studies [169] show that total plasma antioxidant capacity is increased after oral administration of proanthocyanidins. The increased antioxidant capacity decreases the extent of lipid peroxidation [169] and platelet aggregation [170], and reduces oxidation susceptibility of low density lipoproteins (LDLs) [171]. Proanthocyanidins also attenuate ischemic damage to myocardium [172] by neutralizing free radicals [173] and inhibiting apoptotic processes in cardiomyocytes [174]. Moreover, Ramirez and Roa [175] suggested that proanthocyanidins possessed gastroprotective and anti-ulcerogenic effects. Some proanthocyanidins-rich extracts demonstrate additional health effects; for example, those of cranberry juice prevent adhesion of pathogenic bacteria to epithelial cells in the urinary tract [176,177], those found in cinnamon improve the metabolism of glucose and lipids [178], and those of cocoa inhibit diabetes-induced cataract formation [179]. In rats, GSE proanthocyanidins increase bone formation [180] and exert neuroprotective effects in the brain [181], while in mice they attenuate hyperuremia [182]. Animal models also indicate that GSE proanthocyanidins prevent photocarcinogenesis induced by ultraviolet B (UVB) radiation [183], and may inhibit carcinogenesis of breast and colon cancers [184,185]. A study by Yamagishi *et al.* [186] indicates that the scope of anticarcinogenic effects may not be shared by all proanthocyanidins. This study reported that cocoa proanthocyanidins exerted chemopreventive effects in rat lungs, but not in the digestive tract and kidney.

Few studies have been published about effects of proanthocyanidins on glial cells. One of them [187] deals with the effects of a particular proanthocyanidin trimer isolated from a water soluble cinnamon extract. In C6 cells, this trimer attenuated an ischemia-caused increase in intracellular calcium ions, which has been linked to cell swelling. The trimer also attenuated post-ischemic excitotoxicity of glutamate by stimulating its uptake into C6 cells. Other relevant studies analyze the effects of GSE. Roychowdhury *et al.* [188] reported that GSE proanthocyanidins improved viability of rat glial cell cultures subjected to oxidative stress caused by hydrogen peroxide. According to these authors, GSE also stimulated iNOS and therefore increased low-level NO production. The latter claim is controversial since monomeric units of GSE proanthocyanidins include catechin, which has been reported to inhibit NO release [138]. Fujishita *et al.* [189] reported that in primary culture of human astrocytes, GSE proanthocyanidins increased production of IL-6, which was released from the astrocytes as a neuroprotective paracrine and prevented the death of adjacent neurons. However, the mechanisms by which IL-6 protects neurons are also responsible for survival of GBM cells [86].

In the U87 glioblastoma cell line, cranberry juice proanthocyanidins arrest cells in G1 phase of the cell cycle and induce cell death within 48 h of exposure. In animal models, they also slow the growth of explant tumors consisting of U87 cells [190]. This cell line was used by Zhang *et al.* [191] to perform experiments on oligomer procyanidins (DP 2–15) from GSE. The oligomers significantly inhibited growth of glioblastoma cells, induced their cell-cycle arrest and a non-apoptotic cell death, and inhibited their chemotaxis, which has been implied in tumor cell invasion and metastasis. The oligomers also inhibited phosphorylations in the pro-oncogene MAPK signaling cascade. In another study [192], the same authors reported that the oligomers induced ROS production in both U87 cells and rat glioma C6 cells. Their conclusion contravenes the findings of other studies [193,194], which reported antioxidative activity of proanthocyanidins. However, Agarwal *et al.* [145] reported a similar pro-oxidative activity in U87-MG cells treated with EGCG, a catechin derivative. Increase in ROS production is a potential mechanism by which proanthocyanidins inhibit proliferation of GBM cells and induce their death.

## 6. Conclusions

Herbal supplements containing flavonoids have already been introduced into clinical therapy of some non-malignant brain diseases. For instance, *Hypericum perforatum* (Saint John’s wort) extract is used for treatment of major depression. It is safer and at least as effective as selective serotonin reuptake inhibitors, which are the drugs of choice for this condition [195]. The major components of hypericum extract are hypericin (a polycyclic aromatic dianthroquinone), hyperforin (a prenylated phloroglucinol derivative) and various flavonoids including quercetin [196]. While hypericin is unable to pass the BBB in monkeys [197], low concentrations of both hyperforin [198] and quercetin [199] have been detected in rodent brains after oral administration of hypericum extract. These results suggest that flavonoids—alongside hyperforin—contribute to the anti-depressant activity of hypericum.

Supplements with flavonoids have also been used for therapy of malignant diseases outside the brain. PC-SPES, a preparation containing extracts of eight herbs mostly used in Chinese and Ayurvedic traditional medicines, has been used as second-line treatment for progressive androgen-independent prostatic cancer, and as an alternative to conventional anti-androgen therapy in case of hormone-responsive prostatic tumors [200,201]. Its major active components are the phytosterols β-sitosterol and stigmasterol which possess estrogenic activity, and the flavonoids baicalin and licochalcone A [200]. Baicalin induces cell cycle arrest and apoptosis of prostatic cancer cells [202], while licochalcone A acts as a phytoestrogen and modulates the expression of the pro-apoptotic protein Bcl-2 [203]. In clinical trials [201], PC-SPES reduced serum levels of prostate-specific androgen (PSA),a marker of prostatic cancer, in patients with both the androgen-dependent and androgen-independent type of this malignancy, although some patients of the both types subsequently experienced disease progression. In patients with the androgen-dependent type it also reduced testosterone production.

Examples of hypericum and PC-SPES demonstrate that flavonoids can be effectively used in therapy of both brain diseases and cancer, thus their use for GBM prevention and treatment could be envisaged. Reduction of oxidative stress appears to be a common mechanism by which they protect glial cells. Quercetin, catechins and proanthocyanidins all possess antioxidative activity, although proanthocyanidins and EGCG may also act as pro-oxidants. In addition, quercetin and some catechin derivatives, *i.e.*, catechin and EGCG, protect glial cells and adjacent neurons against inflammation, while the catechin derivative ECG and a proanthocyanidin trimer from cranberry juice reduce glutamate-induced excitotoxicity. With respect to GBM cells, most of the discussed flavonoids, *i.e.*, quercetin, EGCG, proanthocyanidins from GSE and cranberry juice, induce the arrest of their cell cycle and eventually their cell death by both apoptotic and non-apoptotic mechanisms. In addition, quercetin and EGCG inhibit the pro-oncogene MAPK signaling pathway, and EGCG also intensifies the effects of both ionizing radiation and chemotherapeutic drugs on GBM cells.

However, most of these effects have been observed only *in vitro* or in animal models, while clinical studies on humans are lacking. The effects observed *in vitro* may not necessarily appear *in vivo* due to low bioavailability of the discussed flavonoids, as well as their limited ability to cross the BBB. The ability of proanthocyanidins to cross the barrier is particularly questionable. Given their limited bioavailability, the intake of flavonoids in normal Western diet is too low to produce brain concentrations in the same range as in the studies that reported their beneficial effects. Therefore, regular use of dietary supplements containing flavonoids is needed to increase systemic and brain concentrations of flavonoids, but even such supplementation may not be sufficient to induce protective and anti-carcinogenic effects.

## References

[1] Louis D.N., Ohgaki H., Wiestler O.D., Cavenee W.K., Burger P.C., Jouvet A., Scheithauer B.W., Kleihues P. (2007). The 2007 who classification of tumours of the central nervous system. Acta Neuropathol..

[2] Johnson D.R., O’Neill B.P. (2012). Glioblastoma survival in the united states before and during the temozolomide era. J. Neurooncol..

[3] Eyler C.E., Rich J.N. (2008). Survival of the fittest: Cancer stem cells in therapeutic resistance and angiogenesis. J. Clin. Oncol..

[4] Reya T., Morrison S.J., Clarke M.F., Weissman I.L. (2001). Stem cells, cancer, and cancer stem cells. Nature.

[5] Eramo A., Ricci-Vitiani L., Zeuner A., Pallini R., Lotti F., Sette G., Pilozzi E., Larocca L.M., Peschle C., de Maria R. (2006). Chemotherapy resistance of glioblastoma stem cells. Cell Death Differ..

[6] Stupp R., Mason W.P., van den Bent M.J., Weller M., Fisher B., Taphoorn M.J., Belanger K., Brandes A.A., Marosi C., Bogdahn U. (2005). Radiotherapy plus concomitant and adjuvant temozolomide for glioblastoma. N. Engl. J. Med..

[7] Bernstein J.J., Woodard C.A. (1995). Glioblastoma cells do not intravasate into blood vessels. Neurosurgery.

[8] Silbergeld D.L., Rostomily R.C., Alvord E.C. (1991). The cause of death in patients with glioblastoma is multifactorial: Clinical factors and autopsy findings in 117 cases of supratentorial glioblastoma in adults. J. Neurooncol..

[9] Rajaraman P., Hutchinson A., Rothman N., Black P.M., Fine H.A., Loeffler J.S., Selker R.G., Shapiro W.R., Linet M.S., Inskip P.D. (2008). Oxidative response gene polymorphisms and risk of adult brain tumors. Neuro Oncol..

[10] Cotelle N. (2001). Role of flavonoids in oxidative stress. Curr. Top. Med. Chem..

[11] Chun O.K., Chung S.J., Song W.O. (2007). Estimated dietary flavonoid intake and major food sources of U.S. adults. J. Nutr..

[12] Treutter D. (2005). Significance of flavonoids in plant resistance and enhancement of their biosynthesis. Plant Biol..

[13] Das D.K. (1994). Naturally occurring flavonoids: Structure, chemistry, and high-performance liquid chromatography methods for separation and characterization. Methods Enzymol..

[14] Manach C., Williamson G., Morand C., Scalbert A., Rémésy C. (2005). Bioavailability and bioefficacy of polyphenols in humans. I. Review of 97 bioavailability studies. Am. J. Clin. Nutr..

[15] Wolffram S., Blöck M., Ader P. (2002). Quercetin-3-glucoside is transported by the glucose carrier SGLT1 across the brush border membrane of rat small intestine. J. Nutr..

[16] Hollman P.C., Katan M.B. (1997). Absorption, metabolism and health effects of dietary flavonoids in man. Biomed. Pharmacother..

[17] Liu Y., Hu M. (2002). Absorption and metabolism of flavonoids in the Caco-2 cell culture model and a perused rat intestinal model. Drug Metab. Dispos..

[18] Youdim K.A., Shukitt-Hale B., Joseph J.A. (2004). Flavonoids and the brain: Interactions at the blood-brain barrier and their physiological effects on the central nervous system. Free Radic. Biol. Med..

[19] Youdim K.A., Dobbie M.S., Kuhnle G., Proteggente A.R., Abbott N.J., Rice-Evans C. (2003). Interaction between flavonoids and the blood-brain barrier: *In vitro* studies. J. Neurochem..

[20] Begley D., Sharma S., Westman J. (2004). Efflux mechanisms in the central nervous system: A powerful influence on drug distribution within the brain. Blood-Spinal Cord and Brain Barriers in Health and Disease.

[21] Youdim K.A., Qaiser M.Z., Begley D.J., Rice-Evans C.A., Abbott N.J. (2004). Flavonoid permeability across an *in situ* model of the blood-brain barrier. Free Radic. Biol. Med..

[22] Borst P., Elferink R.O. (2002). Mammalian abc transporters in health and disease. Annu. Rev. Biochem..

[23] Ishige K., Schubert D., Sagara Y. (2001). Flavonoids protect neuronal cells from oxidative stress by three distinct mechanisms. Free Radic. Biol. Med..

[24] Mandel S., Youdim M.B. (2004). Catechin polyphenols: Neurodegeneration and neuroprotection in neurodegenerative diseases. Free Radic. Biol. Med..

[25] Raso G.M., Meli R., di Carlo G., Pacilio M., di Carlo R. (2001). Inhibition of inducible nitric oxide synthase and cyclooxygenase-2 expression by flavonoids in macrophage J774A.1. Life Sci..

[26] Galati G., O’Brien P.J. (2004). Potential toxicity of flavonoids and other dietary phenolics: Significance for their chemopreventive and anticancer properties. Free Radic. Biol. Med..

[27] Blokhina O., Virolainen E., Fagerstedt K.V. (2003). Antioxidants, oxidative damage and oxygen deprivation stress: A review. Ann. Bot..

[28] Fruehauf J.P., Meyskens F.L. (2007). Reactive oxygen species: A breath of life or death?. Clin. Cancer Res..

[29] Leslie E.M., Mao Q., Oleschuk C.J., Deeley R.G., Cole S.P. (2001). Modulation of multidrug resistance protein 1 (MRP1/ABCC1) transport and atpase activities by interaction with dietary flavonoids. Mol. Pharmacol..

[30] López-Lázaro M., Martín-Cordero C., Toro M.V., Ayuso M.J. (2002). Flavonoids as DNA topoisomerase I poisons. J. Enzyme Inhib. Med. Chem..

[31] Rong Y., Yang E.B., Zhang K., Mack P. (2000). Quercetin-induced apoptosis in the monoblastoid cell line U937 *in vitro* and the regulation of heat shock proteins expression. Anticancer Res..

[32] Kobuchi H., Roy S., Sen C.K., Nguyen H.G., Packer L. (1999). Quercetin inhibits inducible ICAM-1 expression in human endothelial cells through the JNK pathway. Am. J. Physiol..

[33] Ngomuo A.J., Jones R.S. (1996). Cytotoxicity studies of quercetin, shikimate, cyclohexanecarboxylate and ptaquiloside. Vet. Hum. Toxicol..

[34] Yamakoshi J., Saito M., Kataoka S., Kikuchi M. (2002). Safety evaluation of proanthocyanidin-rich extract from grape seeds. Food Chem. Toxicol..

[35] Hodek P., Trefil P., Stiborová M. (2002). Flavonoids-potent and versatile biologically active compounds interacting with cytochromes P450. Chem. Biol. Interact..

[36] Ross J.A. (1998). Maternal diet and infant leukemia: A role for DNA topoisomerase II inhibitors?. Int. J. Cancer Suppl..

[37] Pérez-Coll C.S., Herkovits J. (2004). Lethal and teratogenic effects of naringenin evaluated by means of an amphibian embryo toxicity test (AMPHITOX). Food Chem. Toxicol..

[38] Schönthal A.H. (2011). Adverse effects of concentrated green tea extracts. Mol. Nutr. Food Res..

[39] Choi J.S., Choi Y.J., Shin S.Y., Li J., Kang S.W., Bae J.Y., Kim D.S., Ji G.E., Kang J.S., Kang Y.H. (2008). Dietary flavonoids differentially reduce oxidized LDL-induced apoptosis in human endothelial cells: Role of MAPK- and JAK/STAT-signaling. J. Nutr..

[40] Parker-Athill E., Luo D., Bailey A., Giunta B., Tian J., Shytle R.D., Murphy T., Legradi G., Tan J. (2009). Flavonoids, a prenatal prophylaxis via targeting JAK2/STAT3 signaling to oppose IL-6/MIA associated autism. J. Neuroimmunol..

[41] Virgili F., Acconcia F., Ambra R., Rinna A., Totta P., Marino M. (2004). Nutritional flavonoids modulate estrogen receptor alpha signaling. IUBMB Life.

[42] Lee E.R., Kang Y.J., Kim J.H., Lee H.T., Cho S.G. (2005). Modulation of apoptosis in HaCaT keratinocytes via differential regulation of ERK signaling pathway by flavonoids. J. Biol. Chem..

[43] Fraga C.G., Oteiza P.I. (2011). Dietary flavonoids: Role of (−)-epicatechin and related procyanidins in cell signaling. Free Radic. Biol. Med..

[44] Ruiz P.A., Haller D. (2006). Functional diversity of flavonoids in the inhibition of the proinflammatory NF-κB, IRF, and Akt signaling pathways in murine intestinal epithelial cells. J. Nutr..

[45] Schroeter H., Boyd C., Spencer J.P., Williams R.J., Cadenas E., Rice-Evans C. (2002). MAPK signaling in neurodegeneration: Influences of flavonoids and of nitric oxide. Neurobiol. Aging.

[46] Amado N.G., Fonseca B.F., Cerqueira D.M., Neto V.M., Abreu J.G. (2011). Flavonoids: Potential Wnt/beta-catenin signaling modulators in cancer. Life Sci..

[47] Muthian G., Bright J.J. (2004). Quercetin, a flavonoid phytoestrogen, ameliorates experimental allergic encephalomyelitis by blocking IL-12 signaling through JAK-STAT pathway in T lymphocyte. J. Clin. Immunol..

[48] Krol W., Czuba Z., Scheller S., Paradowski Z., Shani J. (1994). Structure-activity relationship in the ability of flavonols to inhibit chemiluminescence. J. Ethnopharmacol..

[49] Sampson L., Rimm E., Hollman P.C., de Vries J.H., Katan M.B. (2002). Flavonol and flavone intakes in us health professionals. J. Am. Diet Assoc..

[50] Mennen L.I., Walker R., Bennetau-Pelissero C., Scalbert A. (2005). Risks and safety of polyphenol consumption. Am. J. Clin. Nutr..

[51] Sak K. (2014). Site-specific anticancer effects of dietary flavonoid quercetin. Nutr. Cancer.

[52] Aherne S.A., O’Brien N.M. (2002). Dietary flavonols: Chemistry, food content, and metabolism. Nutrition.

[53] Dajas F. (2012). Life or death: Neuroprotective and anticancer effects of quercetin. J. Ethnopharmacol..

[54] Egert S., Wolffram S., Bosy-Westphal A., Boesch-Saadatmandi C., Wagner A.E., Frank J., Rimbach G., Mueller M.J. (2008). Daily quercetin supplementation dose-dependently increases plasma quercetin concentrations in healthy humans. J. Nutr..

[55] Shimoi K., Yoshizumi K., Kido T., Usui Y., Yumoto T. (2003). Absorption and urinary excretion of quercetin, rutin, and alphag-rutin, a water soluble flavonoid, in rats. J. Agric. Food Chem..

[56] Smith A.J., Kavuru P., Wojtas L., Zaworotko M.J., Shytle R.D. (2011). Cocrystals of quercetin with improved solubility and oral bioavailability. Mol. Pharm..

[57] Hollman P.C., de Vries J.H., van Leeuwen S.D., Mengelers M.J., Katan M.B. (1995). Absorption of dietary quercetin glycosides and quercetin in healthy ileostomy volunteers. Am. J. Clin. Nutr..

[58] Boots A.W., Haenen G.R., Bast A. (2008). Health effects of quercetin: From antioxidant to nutraceutical. Eur. J. Pharmacol..

[59] Arts M., Dallinga J., Voss H., Haenen G., Bast A. (2004). A new approach to assess the total antioxidant capacity using the teac assay. Food Chem..

[60] Hirvonen T., Virtamo J., Korhonen P., Albanes D., Pietinen P. (2001). Flavonol and flavone intake and the risk of cancer in male smokers (Finland). Cancer Causes Control.

[61] Knekt P., Kumpulainen J., Järvinen R., Rissanen H., Heliövaara M., Reunanen A., Hakulinen T., Aromaa A. (2002). Flavonoid intake and risk of chronic diseases. Am. J. Clin. Nutr..

[62] Hertog M.G., Feskens E.J., Hollman P.C., Katan M.B., Kromhout D. (1993). Dietary antioxidant flavonoids and risk of coronary heart disease: The zutphen elderly study. Lancet.

[63] MacNee W. (2001). Oxidative stress and lung inflammation in airways disease. Eur. J. Pharmacol..

[64] Rahman I., Gilmour P.S., Jimenez L.A., MacNee W. (2002). Oxidative stress and TNF-α induce histone acetylation and NF-κB/AP-1 activation in alveolar epithelial cells: Potential mechanism in gene transcription in lung inflammation. Mol. Cell Biochem..

[65] Geraets L., Moonen H.J., Brauers K., Wouters E.F., Bast A., Hageman G.J. (2007). Dietary flavones and flavonoles are inhibitors of poly(ADP-ribose)polymerase-1 in pulmonary epithelial cells. J. Nutr..

[66] Bureau G., Longpré F., Martinoli M.G. (2008). Resveratrol and quercetin, two natural polyphenols, reduce apoptotic neuronal cell death induced by neuroinflammation. J. Neurosci. Res..

[67] Ghosh B. (1999). Quercetin inhibits LPS-induced nitric oxide and tumor necrosis factor-α production in murine macrophages. Int. J. Immunopharmacol..

[68] Lee E.S., Lee H.E., Shin J.Y., Yoon S., Moon J.O. (2003). The flavonoid quercetin inhibits dimethylnitrosamine-induced liver damage in rats. J. Pharm. Pharmacol..

[69] Cushnie T.P., Lamb A.J. (2005). Antimicrobial activity of flavonoids. Int. J. Antimicrob. Agents.

[70] Bucki R., Pastore J.J., Giraud F., Sulpice J.C., Janmey P.A. (2003). Flavonoid inhibition of platelet procoagulant activity and phosphoinositide synthesis. J. Thromb. Haemost..

[71] De Whalley C.V., Rankin S.M., Hoult J.R., Jessup W., Leake D.S. (1990). Flavonoids inhibit the oxidative modification of low density lipoproteins by macrophages. Biochem. Pharmacol..

[72] Duarte J., Pérez-Palencia R., Vargas F., Ocete M.A., Pérez-Vizcaino F., Zarzuelo A., Tamargo J. (2001). Antihypertensive effects of the flavonoid quercetin in spontaneously hypertensive rats. Br. J. Pharmacol..

[73] Kuo S.M. (1996). Antiproliferative potency of structurally distinct dietary flavonoids on human colon cancer cells. Cancer Lett..

[74] Gulati N., Laudet B., Zohrabian V.M., Murali R., Jhanwar-Uniyal M. (2006). The antiproliferative effect of quercetin in cancer cells is mediated via inhibition of the PI3K-Akt/PKB pathway. Anticancer Res..

[75] Gitika B., Sai Ram M., Sharma S.K., Ilavazhagan G., Banerjee P.K. (2006). Quercetin protects C6 glial cells from oxidative stress induced by tertiary-butylhydroperoxide. Free Radic. Res..

[76] Chen T.J., Jeng J.Y., Lin C.W., Wu C.Y., Chen Y.C. (2006). Quercetin inhibition of ROS-dependent and -independent apoptosis in rat glioma C6 cells. Toxicology.

[77] Wu B.Y., Yu A.C. (2000). Quercetin inhibits c-fos, heat shock protein, and glial fibrillary acidic protein expression in injured astrocytes. J. Neurosci. Res..

[78] Panickar K.S., Anderson R.A. (2011). Mechanisms underlying the protective effects of myricetin and quercetin following oxygen-glucose deprivation-induced cell swelling and the reduction in glutamate uptake in glial cells. Neuroscience.

[79] Volk C., Kempski B., Kempski O.S. (1997). Inhibition of lactate export by quercetin acidifies rat glial cells *in vitro*. Neurosci. Lett..

[80] Jakubowicz-Gil J., Langner E., Bądziul D., Wertel I., Rzeski W. (2013). Apoptosis induction in human glioblastoma multiforme T98G cells upon temozolomide and quercetin treatment. Tumour Biol..

[81] Braganhol E., Zamin L.L., Canedo A.D., Horn F., Tamajusuku A.S., Wink M.R., Salbego C., Battastini A.M. (2006). Antiproliferative effect of quercetin in the human U138MG glioma cell line. Anticancer Drugs.

[82] Kim E.J., Choi C.H., Park J.Y., Kang S.K., Kim Y.K. (2008). Underlying mechanism of quercetin-induced cell death in human glioma cells. Neurochem. Res..

[83] Siegelin M.D., Reuss D.E., Habel A., Rami A., von Deimling A. (2009). Quercetin promotes degradation of survivin and thereby enhances death-receptor-mediated apoptosis in glioma cells. Neuro Oncol..

[84] Kim H., Moon J.Y., Ahn K.S., Cho S.K. (2013). Quercetin induces mitochondrial mediated apoptosis and protective autophagy in human glioblastoma U373MG cells. Oxid. Med. Cell. Longev..

[85] Jakubowicz-Gil J., Langner E., Wertel I., Piersiak T., Rzeski W. (2010). Temozolomide, quercetin and cell death in the moggccm astrocytoma cell line. Chem. Biol. Interact..

[86] Michaud-Levesque J., Bousquet-Gagnon N., Béliveau R. (2012). Quercetin abrogates IL-6/STAT3 signaling and inhibits glioblastoma cell line growth and migration. Exp. Cell Res..

[87] Amado N.G., Cerqueira D.M., Menezes F.S., da Silva J.F., Neto V.M., Abreu J.G. (2009). Isoquercitrin isolated from hyptis fasciculata reduces glioblastoma cell proliferation and changes beta-catenin cellular localization. Anticancer Drugs.

[88] Siegelin M.D., Reuss D.E., Habel A., Herold-Mende C., von Deimling A. (2008). The flavonoid kaempferol sensitizes human glioma cells to trail-mediated apoptosis by proteasomal degradation of survivin. Mol. Cancer Ther..

[89] Lin C.W., Shen S.C., Chien C.C., Yang L.Y., Shia L.T., Chen Y.C. (2010). 12-*O*-tetradecanoylphorbol-13-acetate-induced invasion/migration of glioblastoma cells through activating PKCα/ERK/NF-κB-dependent MMP-9 expression. J. Cell. Physiol..

[90] Das A., Banik N.L., Ray S.K. (2010). Flavonoids activated caspases for apoptosis in human glioblastoma T98G and U87MG cells but not in human normal astrocytes. Cancer.

[91] Lin Y.C., Hung C.M., Tsai J.C., Lee J.C., Chen Y.L., Wei C.W., Kao J.Y., Way T.D. (2010). Hispidulin potently inhibits human glioblastoma multiforme cells through activation of AMP-activated protein kinase (AMPK). J. Agric. Food Chem..

[92] Poncet-Legrand C., Edelmann A., Putaux J., Cartalade D., Sarni-Manchado P., Vernhet A. (2006). Poly(l-proline) interactions with flavan-3-ols units: Influence of the molecular structure and the polyphenol/protein ratio. Food Hydrocolloids.

[93] Hashimoto F., Ono M., Masuoka C., Ito Y., Sakata Y., Shimizu K., Nonaka G., Nishioka I., Nohara T. (2003). Evaluation of the anti-oxidative effect (*in vitro*) of tea polyphenols. Biosci. Biotechnol. Biochem..

[94] Zhou Z.H., Zhang Y.J., Xu M., Yang C.R. (2005). Puerins A and B, two new 8-C substituted flavan-3-ols from pu-er tea. J. Agric. Food Chem..

[95] Graham H.N. (1992). Green tea composition, consumption, and polyphenol chemistry. Prev. Med..

[96] Arts I.C., Hollman P.C., Kromhout D. (1999). Chocolate as a source of tea flavonoids. Lancet.

[97] Auger C., Al-Awwadi N., Bornet A., Rouanet J., Gasc F., Cros G., Teissedre P. (2004). Catechins and procyanidins in mediterranean diets. Food Res. Int..

[98] De Pascual-Teresa S., Santos-Buelga C., Rivas-Gonzalo J.C. (2000). Quantitative analysis of flavan-3-ols in Spanish foodstuffs and beverages. J. Agric. Food Chem..

[99] Carando S., Teissedre P.L. (1999). Catechin and procyanidin levels in french wines: Contribution to dietary intake. Basic Life Sci..

[100] Seeram N.P., Henning S.M., Niu Y., Lee R., Scheuller H.S., Heber D. (2006). Catechin and caffeine content of green tea dietary supplements and correlation with antioxidant capacity. J. Agric. Food Chem..

[101] Zhu M., Chen Y., Li R.C. (2000). Oral absorption and bioavailability of tea catechins. Planta Med..

[102] Warden B.A., Smith L.S., Beecher G.R., Balentine D.A., Clevidence B.A. (2001). Catechins are bioavailable in men and women drinking black tea throughout the day. J. Nutr..

[103] Baba S., Osakabe N., Natsume M., Muto Y., Takizawa T., Terao J. (2001). *In vivo* comparison of the bioavailability of (+)-catechin, (−)-epicatechin and their mixture in orally administered rats. J. Nutr..

[104] Nakagawa K., Okuda S., Miyazawa T. (1997). Dose-dependent incorporation of tea catechins, (−)-epigallocatechin-3-gallate and (−)-epigallocatechin, into human plasma. Biosci. Biotechnol. Biochem..

[105] Vaidyanathan J.B., Walle T. (2003). Cellular uptake and efflux of the tea flavonoid (−)-epicatechin-3-gallate in the human intestinal cell line Caco-2. J. Pharmacol. Exp. Ther..

[106] Faria A., Pestana D., Teixeira D., Couraud P.O., Romero I., Weksler B., de Freitas V., Mateus N., Calhau C. (2011). Insights into the putative catechin and epicatechin transport across blood-brain barrier. Food Funct..

[107] Wu L., Zhang Q.L., Zhang X.Y., Lv C., Li J., Yuan Y., Yin F.X. (2012). Pharmacokinetics and blood-brain barrier penetration of (+)-catechin and (−)-epicatechin in rats by microdialysis sampling coupled to high-performance liquid chromatography with chemiluminescence detection. J. Agric. Food Chem..

[108] Abd El Mohsen M.M., Kuhnle G., Rechner A.R., Schroeter H., Rose S., Jenner P., Rice-Evans C.A. (2002). Uptake and metabolism of epicatechin and its access to the brain after oral ingestion. Free Radic. Biol. Med..

[109] Yokozawa T., Nakagawa T., Kitani K. (2002). Antioxidative activity of green tea polyphenol in cholesterol-fed rats. J. Agric. Food Chem..

[110] Skrzydlewska E., Ostrowska J., Farbiszewski R., Michalak K. (2002). Protective effect of green tea against lipid peroxidation in the rat liver, blood serum and the brain. Phytomedicine.

[111] Chen L., Lee M.J., Li H., Yang C.S. (1997). Absorption, distribution, elimination of tea polyphenols in rats. Drug Metab. Dispos..

[112] Crespy V., Williamson G. (2004). A review of the health effects of green tea catechins in *in vivo* animal models. J. Nutr..

[113] Jia X.D., Han C. (2000). Chemoprevention of tea on colorectal cancer induced by dimethylhydrazine in wistar rats. World J. Gastroenterol..

[114] Jia X., Han C. (2001). Effects of green tea on colonic aberrant crypt foci and proliferative indexes in rats. Nutr. Cancer.

[115] Matsumoto H., Yamane T., Inagake M., Nakatani H., Iwata Y., Takahashi T., Nishimura H., Nishino H., Nakagawa K., Miyazawa T. (1996). Inhibition of mucosal lipid hyperoxidation by green tea extract in 1,2-dimethylhydrazine-induced rat colonic carcinogenesis. Cancer Lett..

[116] Li N., Han C., Chen J. (1999). Tea preparations protect against DMBA-induced oral carcinogenesis in hamsters. Nutr. Cancer.

[117] Cao J., Xu Y., Chen J., Klaunig J.E. (1996). Chemopreventive effects of green and black tea on pulmonary and hepatic carcinogenesis. Fundam. Appl. Toxicol..

[118] Hirose M., Hasegawa R., Kimura J., Akagi K., Yoshida Y., Tanaka H., Miki T., Satoh T., Wakabayashi K., Ito N. (1995). Inhibitory effects of 1-*O*-hexyl-2,3,5-trimethylhydroquinone (HTHQ), green tea catechins and other antioxidants on 2-amino-6-methyldipyrido[1,2-*a*: 3′,2′-*d*]imidazole (Glu-P-1)-induced rat hepatocarcinogenesis and dose-dependent inhibition by HTHQ of lesion induction by Glu-P-1 or 2-amino-3,8-dimethylimidazo[4,5-*f*]quinoxaline (MeIQx). Carcinogenesis.

[119] Tanaka H., Hirose M., Kawabe M., Sano M., Takesada Y., Hagiwara A., Shirai T. (1997). Post-initiation inhibitory effects of green tea catechins on 7,12-dimethylbenz[*a*]anthracene-induced mammary gland carcinogenesis in female Sprague-Dawley rats. Cancer Lett..

[120] Kavanagh K.T., Hafer L.J., Kim D.W., Mann K.K., Sherr D.H., Rogers A.E., Sonenshein G.E. (2001). Green tea extracts decrease carcinogen-induced mammary tumor burden in rats and rate of breast cancer cell proliferation in culture. J. Cell. Biochem..

[121] Gupta S., Hastak K., Ahmad N., Lewin J.S., Mukhtar H. (2001). Inhibition of prostate carcinogenesis in tramp mice by oral infusion of green tea polyphenols. Proc. Natl. Acad. Sci. USA.

[122] Xu Y., Ho C.T., Amin S.G., Han C., Chung F.L. (1992). Inhibition of tobacco-specific nitrosamine-induced lung tumorigenesis in A/J mice by green tea and its major polyphenol as antioxidants. Cancer Res..

[123] Sazuka M., Murakami S., Isemura M., Satoh K., Nukiwa T. (1995). Inhibitory effects of green tea infusion on *in vitro* invasion and *in vivo* metastasis of mouse lung carcinoma cells. Cancer Lett..

[124] Zhu M., Gong Y., Yang Z., Ge G., Han C., Chen J. (1999). Green tea and its major components ameliorate immune dysfunction in mice bearing lewis lung carcinoma and treated with the carcinogen nnk. Nutr. Cancer.

[125] Zhang G., Miura Y., Yagasaki K. (2002). Effects of dietary powdered green tea and theanine on tumor growth and endogenous hyperlipidemia in hepatoma-bearing rats. Biosci. Biotechnol. Biochem..

[126] Sai K., Kai S., Umemura T., Tanimura A., Hasegawa R., Inoue T., Kurokawa Y. (1998). Protective effects of green tea on hepatotoxicity, oxidative dna damage and cell proliferation in the rat liver induced by repeated oral administration of 2-nitropropane. Food Chem. Toxicol..

[127] Hasegawa R., Chujo T., Sai-Kato K., Umemura T., Tanimura A., Kurokawa Y. (1995). Preventive effects of green tea against liver oxidative dna damage and hepatotoxicity in rats treated with 2-nitropropane. Food Chem. Toxicol..

[128] Rhee S.J., Kim M.J., Kwag O.G. (2002). Effects of green tea catechin on prostaglandin synthesis of renal glomerular and renal dysfunction in streptozotocin-induced diabetic rats. Asia Pac. J. Clin. Nutr..

[129] Rhee S.J., Choi J.H., Park M.R. (2002). Green tea catechin improves microsomal phospholipase A2 activity and the arachidonic acid cascade system in the kidney of diabetic rats. Asia Pac. J. Clin. Nutr..

[130] Yang J.A., Choi J.H., Rhee S.J. (1999). Effects of green tea catechin on phospholipase A2 activity and antithrombus in streptozotocin diabetic rats. J. Nutr. Sci. Vitaminol..

[131] Spencer J.P. (2009). Flavonoids and brain health: Multiple effects underpinned by common mechanisms. Genes Nutr..

[132] Spencer J.P. (2007). The interactions of flavonoids within neuronal signalling pathways. Genes Nutr..

[133] Huang Q., Wu L.J., Tashiro S., Gao H.Y., Onodera S., Ikejima T. (2005). (+)-catechin, an ingredient of green tea, protects murine microglia from oxidative stress-induced dna damage and cell cycle arrest. J. Pharmacol. Sci..

[134] Li R., Huang Y.G., Fang D., Le W.D. (2004). (−)-epigallocatechin gallate inhibits lipopolysaccharide-induced microglial activation and protects against inflammation-mediated dopaminergic neuronal injury. J. Neurosci. Res..

[135] Park I.J., Lee Y.K., Hwang J.T., Kwon D.Y., Ha J., Park O.J. (2009). Green tea catechin controls apoptosis in colon cancer cells by attenuation of H_2_O_2_-stimulated COX-2 expression via the ampk signaling pathway at low-dose H_2_O_2_. Ann. N. Y. Acad. Sci..

[136] Noreen Y., Serrano G., Perera P., Bohlin L. (1998). Flavan-3-ols isolated from some medicinal plants inhibiting COX-1 and COX-2 catalysed prostaglandin biosynthesis. Planta Med..

[137] Mazzio E.A., Harris N., Soliman K.F. (1998). Food constituents attenuate monoamine oxidase activity and peroxide levels in C6 astrocyte cells. Planta Med..

[138] Soliman K.F., Mazzio E.A. (1998). *In vitro* attenuation of nitric oxide production in C6 astrocyte cell culture by various dietary compounds. Proc. Soc. Exp. Biol. Med..

[139] Panickar K.S., Polansky M.M., Anderson R.A. (2009). Green tea polyphenols attenuate glial swelling and mitochondrial dysfunction following oxygen-glucose deprivation in cultures. Nutr. Neurosci..

[140] Abib R.T., Quincozes-Santos A., Nardin P., Wofchuk S.T., Perry M.L., Gonçalves C.A., Gottfried C. (2008). Epicatechin gallate increases glutamate uptake and S100B secretion in C6 cell lineage. Mol. Cell. Biochem..

[141] Shervington A., Pawar V., Menon S., Thakkar D., Patel R. (2009). The sensitization of glioma cells to cisplatin and tamoxifen by the use of catechin. Mol. Biol. Rep..

[142] Chen T.C., Wang W., Golden E.B., Thomas S., Sivakumar W., Hofman F.M., Louie S.G., Schönthal A.H. (2011). Green tea epigallocatechin gallate enhances therapeutic efficacy of temozolomide in orthotopic mouse glioblastoma models. Cancer Lett..

[143] Li H., Li Z., Xu Y.M., Wu Y., Yu K.K., Zhang C., Ji Y.H., Ding G., Chen F.X. (2014). Epigallocatechin-3-gallate induces apoptosis, inhibits proliferation and decreases invasion of glioma cell. Neurosci. Bull..

[144] McLaughlin N., Annabi B., Bouzeghrane M., Temme A., Bahary J.P., Moumdjian R., Béliveau R. (2006). The survivin-mediated radioresistant phenotype of glioblastomas is regulated by RhoA and inhibited by the green tea polyphenol (−)-epigallocatechin-3-gallate. Brain Res..

[145] Agarwal A., Sharma V., Tewari R., Koul N., Joseph C., Sen E. (2008). Epigallocatechin-3-gallate exhibits anti-tumor effect by perturbing redox homeostasis, modulating the release of pro-inflammatory mediators and decreasing the invasiveness of glioblastoma cells. Mol. Med. Rep..

[146] McLaughlin N., Annabi B., Lachambre M.P., Kim K.S., Bahary J.P., Moumdjian R., Béliveau R. (2006). Combined low dose ionizing radiation and green tea-derived epigallocatechin-3-gallate treatment induces human brain endothelial cells death. J. Neurooncol..

[147] Porter L., Harborne J. (1994). Flavans and proanthocyanidins. The Flavonoids—Advances in Research Since 1986.

[148] Santos-Buelga C., Scalbert A. (2000). Proanthocyanidins and tannin-like compounds—Nature, occurrence, dietary intake and effects on nutrition and health. J. Sci. Food Agric..

[149] Gu L., Kelm M.A., Hammerstone J.F., Beecher G., Holden J., Haytowitz D., Gebhardt S., Prior R.L. (2004). Concentrations of proanthocyanidins in common foods and estimations of normal consumption. J. Nutr..

[150] Rasmussen S.E., Frederiksen H., Struntze Krogholm K., Poulsen L. (2005). Dietary proanthocyanidins: Occurrence, dietary intake, bioavailability, and protection against cardiovascular disease. Mol. Nutr. Food Res..

[151] Wilmouth R.C., Turnbull J.J., Welford R.W., Clifton I.J., Prescott A.G., Schofield C.J. (2002). Structure and mechanism of anthocyanidin synthase from arabidopsis thaliana. Structure.

[152] Gu L., Kelm M.A., Hammerstone J.F., Beecher G., Holden J., Haytowitz D., Prior R.L. (2003). Screening of foods containing proanthocyanidins and their structural characterization using LC-MS/MS and thiolytic degradation. J. Agric. Food Chem..

[153] Oliff H. (2007). Scientific and Clinical Monograph for Pycnogenol.

[154] Rohdewald P. (2002). A review of the French maritime pine bark extract (Pycnogenol), a herbal medication with a diverse clinical pharmacology. Int. J. Clin. Pharmacol. Ther..

[155] Shrikhande A. (2000). Wine by-products with health benefits. Food Res. Int..

[156] Rios L.Y., Bennett R.N., Lazarus S.A., Rémésy C., Scalbert A., Williamson G. (2002). Cocoa procyanidins are stable during gastric transit in humans. Am. J. Clin. Nutr..

[157] Déprez S., Brezillon C., Rabot S., Philippe C., Mila I., Lapierre C., Scalbert A. (2000). Polymeric proanthocyanidins are catabolized by human colonic microflora into low-molecular-weight phenolic acids. J. Nutr..

[158] Gonthier M.P., Donovan J.L., Texier O., Felgines C., Remesy C., Scalbert A. (2003). Metabolism of dietary procyanidins in rats. Free Radic. Biol. Med..

[159] Rios L.Y., Gonthier M.P., Rémésy C., Mila I., Lapierre C., Lazarus S.A., Williamson G., Scalbert A. (2003). Chocolate intake increases urinary excretion of polyphenol-derived phenolic acids in healthy human subjects. Am. J. Clin. Nutr..

[160] Düweler K.G., Rohdewald P. (2000). Urinary metabolites of french maritime pine bark extract in humans. Pharmazie.

[161] Deprez S., Mila I., Huneau J.F., Tome D., Scalbert A. (2001). Transport of proanthocyanidin dimer, trimer, and polymer across monolayers of human intestinal epithelial Caco-2 cells. Antioxid. Redox Signal..

[162] Spencer J.P., Schroeter H., Shenoy B., Srai S.K., Debnam E.S., Rice-Evans C. (2001). Epicatechin is the primary bioavailable form of the procyanidin dimers B2 and B5 after transfer across the small intestine. Biochem. Biophys. Res. Commun..

[163] Baba S., Osakabe N., Natsume M., Terao J. (2002). Absorption and urinary excretion of procyanidin B2 [epicatechin-(4β-8)-epicatechin] in rats. Free Radic. Biol. Med..

[164] Donovan J., Manach C., Rios L., Morand C., Scalbert A., Remesy C. (2002). Procyanidins are not bioavailable in rats fed a single meal containing a grapeseed extract or the procyanidin dimer B3. Br. J. Nutr..

[165] Cahn J., Borzeix M.G. (1983). Administration of procyanidolic oligomers in rats. Observed effects on changes in the permeability of the blood-brain barrier. Sem. Hop..

[166] Robert A.M., Tixier J.M., Robert L., Legeais J.M., Renard G. (2001). Effect of procyanidolic oligomers on the permeability of the blood-brain barrier. Pathol. Biol..

[167] Prasain J.K., Peng N., Dai Y., Moore R., Arabshahi A., Wilson L., Barnes S., Michael Wyss J., Kim H., Watts R.L. (2009). Liquid chromatography tandem mass spectrometry identification of proanthocyanidins in rat plasma after oral administration of grape seed extract. Phytomedicine.

[168] Prior R.L., Gu L. (2005). Occurrence and biological significance of proanthocyanidins in the american diet. Phytochemistry.

[169] Natella F., Belelli F., Gentili V., Ursini F., Scaccini C. (2002). Grape seed proanthocyanidins prevent plasma postprandial oxidative stress in humans. J. Agric. Food Chem..

[170] Murphy K.J., Chronopoulos A.K., Singh I., Francis M.A., Moriarty H., Pike M.J., Turner A.H., Mann N.J., Sinclair A.J. (2003). Dietary flavanols and procyanidin oligomers from cocoa (*Theobroma cacao*) inhibit platelet function. Am. J. Clin. Nutr..

[171] Wan Y., Vinson J.A., Etherton T.D., Proch J., Lazarus S.A., Kris-Etherton P.M. (2001). Effects of cocoa powder and dark chocolate on LDL oxidative susceptibility and prostaglandin concentrations in humans. Am. J. Clin. Nutr..

[172] Facino R.M., Carini M., Aldini G., Berti F., Rossoni G., Bombardelli E., Morazzoni P. (1999). Diet enriched with procyanidins enhances antioxidant activity and reduces myocardial post-ischaemic damage in rats. Life Sci..

[173] Pataki T., Bak I., Kovacs P., Bagchi D., Das D.K., Tosaki A. (2002). Grape seed proanthocyanidins improved cardiac recovery during reperfusion after ischemia in isolated rat hearts. Am. J. Clin. Nutr..

[174] Sato M., Bagchi D., Tosaki A., Das D.K. (2001). Grape seed proanthocyanidin reduces cardiomyocyte apoptosis by inhibiting ischemia/reperfusion-induced activation of JNK-1 and C-JUN. Free Radic. Biol. Med..

[175] Ramirez R.O., Roa C.C. (2003). The gastroprotective effect of tannins extracted from duhat (*Syzygium cumini* Skeels) bark on HCl/ethanol induced gastric mucosal injury in Sprague-Dawley rats. Clin. Hemorheol. Microcirc..

[176] Foo L.Y., Lu Y., Howell A.B., Vorsa N. (2000). The structure of cranberry proanthocyanidins which inhibit adherence of uropathogenic P-fimbriated escherichia coli *in vitro*. Phytochemistry.

[177] Foo L.Y., Lu Y., Howell A.B., Vorsa N. (2000). A-type proanthocyanidin trimers from cranberry that inhibit adherence of uropathogenic P-fimbriated escherichia coli. J. Nat. Prod..

[178] Khan A., Safdar M., Ali Khan M.M., Khattak K.N., Anderson R.A. (2003). Cinnamon improves glucose and lipids of people with type 2 diabetes. Diabetes Care.

[179] Osakabe N., Yamagishi M., Natsume M., Yasuda A., Osawa T. (2004). Ingestion of proanthocyanidins derived from cacao inhibits diabetes-induced cataract formation in rats. Exp. Biol. Med..

[180] Kamitani Y., Maki K., Tofani I., Nishikawa Y., Tsukamoto K., Kimura M. (2004). Effects of grape seed proanthocyanidins extract on mandibles in developing rats. Oral Dis..

[181] Deshane J., Chaves L., Sarikonda K.V., Isbell S., Wilson L., Kirk M., Grubbs C., Barnes S., Meleth S., Kim H. (2004). Proteomics analysis of rat brain protein modulations by grape seed extract. J. Agric. Food. Chem..

[182] Wang Y., Zhu J.X., Kong L.D., Yang C., Cheng C.H., Zhang X. (2004). Administration of procyanidins from grape seeds reduces serum uric acid levels and decreases hepatic xanthine dehydrogenase/oxidase activities in oxonate-treated mice. Basic Clin. Pharmacol. Toxicol..

[183] Mittal A., Elmets C.A., Katiyar S.K. (2003). Dietary feeding of proanthocyanidins from grape seeds prevents photocarcinogenesis in SKH-1 hairless mice: Relationship to decreased fat and lipid peroxidation. Carcinogenesis.

[184] Singletary K.W., Meline B. (2001). Effect of grape seed proanthocyanidins on colon aberrant crypts and breast tumors in a rat dual-organ tumor model. Nutr. Cancer.

[185] Gali-Muhtasib H.U., Younes I.H., Karchesy J.J., El-Sabban M.E. (2001). Plant tannins inhibit the induction of aberrant crypt foci and colonic tumors by 1,2-dimethylhydrazine in mice. Nutr. Cancer.

[186] Yamagishi M., Natsume M., Osakabe N., Okazaki K., Furukawa F., Imazawa T., Nishikawa A., Hirose M. (2003). Chemoprevention of lung carcinogenesis by cacao liquor proanthocyanidins in a male rat multi-organ carcinogenesis model. Cancer Lett..

[187] Panickar K.S., Polansky M.M., Graves D.J., Urban J.F., Anderson R.A. (2012). A procyanidin type a trimer from cinnamon extract attenuates glial cell swelling and the reduction in glutamate uptake following ischemia-like injury *in vitro*. Neuroscience.

[188] Roychowdhury S., Wolf G., Keilhoff G., Bagchi D., Horn T. (2001). Protection of primary glial cells by grape seed proanthocyanidin extract against nitrosative/oxidative stress. Nitric Oxide.

[189] Fujishita K., Ozawa T., Shibata K., Tanabe S., Sato Y., Hisamoto M., Okuda T., Koizumi S. (2009). Grape seed extract acting on astrocytes reveals neuronal protection against oxidative stress via interleukin-6-mediated mechanisms. Cell. Mol. Neurobiol..

[190] Ferguson P.J., Kurowska E.M., Freeman D.J., Chambers A.F., Koropatnick J. (2006). *In vivo* inhibition of growth of human tumor lines by flavonoid fractions from cranberry extract. Nutr. Cancer.

[191] Zhang F.J., Yang J.Y., Mou Y.H., Sun B.S., Ping Y.F., Wang J.M., Bian X.W., Wu C.F. (2009). Inhibition of U-87 human glioblastoma cell proliferation and formyl peptide receptor function by oligomer procyanidins (F2) isolated from grape seeds. Chem. Biol. Interact..

[192] Zhang F., Yang J., Mou Y., Sun B., Wang J., Wang F., Wu C. (2009). Oligomeric procyanidins induce generation of reactive oxygen species and collapse of mitochondrial membrane potential in glioblastoma cell lines. Chin. Herb. Med..

[193] Lotito S.B., Actis-Goretta L., Renart M.L., Caligiuri M., Rein D., Schmitz H.H., Steinberg F.M., Keen C.L., Fraga C.G. (2000). Influence of oligomer chain length on the antioxidant activity of procyanidins. Biochem. Biophys. Res. Commun..

[194] Verstraeten S.V., Keen C.L., Schmitz H.H., Fraga C.G., Oteiza P.I. (2003). Flavan-3-ols and procyanidins protect liposomes against lipid oxidation and disruption of the bilayer structure. Free Radic. Biol. Med..

[195] Szegedi A., Kohnen R., Dienel A., Kieser M. (2005). Acute treatment of moderate to severe depression with hypericum extract WS 5570 (St John’s wort): Randomised controlled double blind non-inferiority trial *versus* paroxetine. BMJ.

[196] Silva B., Ferreres F., Malva O., Dias A. (2005). Phytochemical and antioxidant characterization of *Hypericum perforatum* alcoholic extracts. Food Chem..

[197] Fox E., Murphy R.F., McCully C.L., Adamson P.C. (2001). Plasma pharmacokinetics and cerebrospinal fluid penetration of hypericin in nonhuman primates. Cancer Chemother. Pharmacol..

[198] Keller J.H., Karas M., Müller W.E., Volmer D.A., Eckert G.P., Tawab M.A., Blume H.H., Dingermann T., Schubert-Zsilavecz M. (2003). Determination of hyperforin in mouse brain by high-performance liquid chromatography/tandem mass spectrometry. Anal. Chem..

[199] Paulke A., Schubert-Zsilavecz M., Wurglics M. (2006). Determination of St. John’s wort flavonoid-metabolites in rat brain through high performance liquid chromatography coupled with fluorescence detection. J. Chromatogr. B Anal. Technol. Biomed. Life Sci..

[200] Sovak M., Seligson A.L., Konas M., Hajduch M., Dolezal M., Machala M., Nagourney R. (2002). Herbal composition PC-SPES for management of prostate cancer: Identification of active principles. J. Natl. Cancer Inst..

[201] Small E.J., Frohlich M.W., Bok R., Shinohara K., Grossfeld G., Rozenblat Z., Kelly W.K., Corry M., Reese D.M. (2000). Prospective trial of the herbal supplement PC-SPES in patients with progressive prostate cancer. J. Clin. Oncol..

[202] Ikezoe T., Chen S.S., Heber D., Taguchi H., Koeffler H.P. (2001). Baicalin is a major component of PC-SPES which inhibits the proliferation of human cancer cells via apoptosis and cell cycle arrest. Prostate.

[203] Rafi M.M., Rosen R.T., Vassil A., Ho C.T., Zhang H., Ghai G., Lambert G., DiPaola R.S. (2000). Modulation of bcl-2 and cytotoxicity by licochalcone-A, a novel estrogenic flavonoid. Anticancer Res..

